# Genes related to antiviral activity are differentially expressed in CD4^+^ T cell in HAM/TSP patients

**DOI:** 10.1186/1742-4690-11-S1-P75

**Published:** 2014-01-07

**Authors:** Mariana T Pinto, Tathiane M Malta, Daniel G Pinheiro, Evandra S Rodrigues, Rodrigo A Panepucci, Alessandra P Sousa, Kelen CR Malmegrim, Patricia VB Palma, Osvaldo M Takayanagui, Dimas T Covas, Simone Kashima

**Affiliations:** 1 Regional Blood Center of Ribeirão Preto, University of São Paulo (USP), Brazil; 2 Faculty of Pharmaceutical Sciences of Ribeirão Preto, USP; 3 Faculty of Medicine of Ribeirão Preto, USP, Brazil

## 

CD4^+^ T cells play a central role in HTLV-1 infection. We investigated the global gene expression profile of circulating CD4^+^ T cells in HTLV-1-infected individuals. The microarray platform was performed using 12 individual RNA samples: healthy control (CT, n=4), asymptomatic HTLV-1 carrier (HAC, n=4) and HAM/TSP group (n=4). Proviral load (PVL), Tax expression and the percentage of CD4^+^Foxp3^+^ cells were analyzed. Hierarchical clustering analysis showed that CT and HTLV-infected groups clustered separately. We identified 25 differentially expressed genes in common between CT *vs*. HAM/TSP and HAM/TSP *vs.* HAC analyses and we observed their participation in granzyme A (GZMA) signaling pathways. GZMA, GZMB and PRF1 were validated by qRT-PCR. GZMA and PRF1 gene expression was significantly increased in HAM/TSP group compared to CT and HAC groups. No difference was observed in gene expression level of GZMB. Regulatory T cells (Treg) cells have cytolytic capacity and perforin/granzyme pathways are required for this activity. Foxp3 gene expression was evaluated and it was significantly increased in HAM/TSP group compared to CT and HAC groups. GZMA, GZMB, and PRF1 genes were positively correlated to Foxp3 gene expression. PRF1 and Foxp3 genes were positively correlated with Tax expression and PVL. The percentage of CD4^+^Foxp3^+^ cells revealed a significant increase in HAC and HAM/TSP groups compared to CT. Our results suggest that Treg cells may use the perforin/granzyme pathway as a system to suppress the immune cells and may contribute to immunodeficiency, which is observed in HTLV-1 infection.

## Financial support

FUNDHERP, CTC, INCTC, FAPESP.

